# Do you believe in the power of clinical examination? The answer must be yes!

**DOI:** 10.1590/S1516-31802003000600001

**Published:** 2003-11-06

**Authors:** Isabela M. Benseñor

Dinosaurs were declared extinct many centuries ago. Mankind cannot now do anything to undo this for itself. In an article in 1996, Schechter et al. say in their first line: "Skilled history-taking is in danger of becoming a lost art". Mankind, and doctors especially, can and must do their best to prevent this extinction.^1^

Hampton et al., in 1975, stated that history-taking was responsible for 82.5% of all diagnoses in their group of 80 patients.^2^ Physical examination was responsible for 8.75% and laboratory investigation for a further 8.75%. Sandler, in 1979, analyzed the importance of history-taking, examination and routine and special investigation among 630 patients.^3^ He concluded that history-taking was the most important factor in both diagnosis and management in cardiovascular, neurological, respiratory, urinary and other miscellaneous problems, and was decisive in 56% of all diagnoses. Hampton's and Sandler's data were obtained from British general practitioners.

Peterson et al. attempted to quantify the relative contributions of history-taking, physical examination and laboratory investigation in making medical diagnoses in an outpatient unit in the United States in 1992.^4^ They concluded that history-taking was responsible for the final diagnosis in 76% of all patients. Physical examination led to the diagnosis in another 12%, and laboratory investigation in a further 11% of all patients. They also concluded that, although physical examination and laboratory investigation led to fewer diagnoses, they were instrumental in excluding certain diagnostic possibilities and increasing physicians’ confidence in their diagnoses.

More recently, in India in 2000, Roshan and Rao attempted to quantify the relative contributions of history-taking, physical examination and laboratory investigations in making medical diagnoses.^5^ History-taking was responsible for the diagnoses of 78.6% of all patients. Physical examination was responsible for another 8.2% and laboratory investigation a further 13.2% of all diagnoses.

Now, recent research performed in Brazil has shown that, after excluding dermatological diagnoses and diagnoses from screening, history-taking is responsible for 77.8% of all diagnoses, physical examination for 10% and laboratory investigation for a further 10% (Benseñor IM, unpublished data). If we take diagnoses from screening into consideration, the accuracy of history-taking will be decreased. However, it must be remembered that diagnoses from screening are made in relation to asymptomatic people!

What do such data tell us? They show that the most important tests we have ever devised for obtaining clinical diagnoses are history-taking and physical examination! We now have a lot of invasive and non-invasive complementary investigations, many of them extremely expensive, but the most powerful tool that physicians have is still the contact between them and the patient during clinical examination. So, if you want to improve your diagnostic capability, the best thing we can do is to "take" a good history or, choosing a better word, "construct" a good history. The choice of word is significant since, as previously defined by Platt and Platt, "taking" a history implies an aggressor acting on a victim, whereas "building" a history allows for a joint effort between the doctor and the patient.^6^ Let us do this!
